# Association Between *NPHS2* p.R229Q and Focal Segmental Glomerular Sclerosis/Steroid-Resistant Nephrotic Syndrome

**DOI:** 10.3389/fmed.2022.937122

**Published:** 2022-07-22

**Authors:** Qiongxiu Zhou, Qinjie Weng, Xiaoyan Zhang, Yunzi Liu, Jun Tong, Xu Hao, Hao Shi, Pingyan Shen, Hong Ren, Jingyuan Xie, Nan Chen

**Affiliations:** ^1^Department of Nephrology, Institute of Nephrology, Ruijin Hospital, School of Medicine, Shanghai Jiao Tong University, Shanghai, China; ^2^Department of Nephrology, The First Affiliated Hospital of Wenzhou Medical University, Wenzhou, China

**Keywords:** focal segmental glomerular sclerosis, *NPHS2*, p.R229Q, steroid resistant nephrotic syndrome, meta-analysis

## Abstract

**Aim:**

*NPHS2* is the coding gene of podocin. This study aims to investigate the association between *NPHS2* p.R229Q (rs61747728), the most frequently reported missense variant of *NPHS2*, and focal segmental glomerular sclerosis (FSGS) or steroid-resistant nephrotic syndrome (SRNS) based on typing the variant in a Chinese FSGS/SRNS cohort and conducting a meta-analysis.

**Method:**

We recruited patients with FSGS or SRNS and healthy individuals. To conduct a meta-analysis, all studies on p.R229Q and FSGS/SRNS were searched from public databases.

**Results:**

In total, we enrolled 204 patients with FSGS, 61 patients with SRNS [46 with FSGS, 9 with minimal change disease (MCD), and six patients with IgA nephropathy (IgAN)], and 100 healthy controls. Unexpectedly, p.R229Q was absent in the patients from our cohort. By meta-analysis of 21 studies including 2,489 patients with FSGS/SRNS and 6,004 healthy controls, we confirmed that the A allele of p.R229Q was significantly associated with increased risk of FSGS/SRNS (allelic OR = 1.9, 95% CI = 1.44-2.52, *P* < 0.001). However, the subgroup analysis showed that the association between p.R229Q and FSGS/SRNS was true only in Caucasians (allelic OR = 2.14, 95%CI = 1.54-2.98, *P* < 0.001) and in early-onset patients (allelic OR: 2.13, 95% CI = 1.21-3.76, *P* = 0.009).

**Conclusion:**

*NPHS2* p.R229Q may play an important role in enhancing the susceptibility of FSGS/SRNS, especially in ethnicity of Caucasian and age of early-onset patients.

## Introduction

Idiopathic nephrotic syndrome (INS) is clinically characterized by edema, massive proteinuria, hypoalbuminemia, and hyperlipidemia. Although steroid is widely used for treatment of INS, about 10-20% of children (1) and 50% of adults patients (2) have steroid-resistant nephrotic syndrome (SRNS). Among different pathologic types of SRNS, focal segmental glomerulosclerosis (FSGS) is the most common cause in children (3), accounting for 60-70% (4, 5), and it also frequently occurs in adults (6).

Over the past decades, dozens of podocyte-related genes had been identified in several monogenic forms of hereditary FSGS/SRNS (7–9). Among these genes, mutations in podocin (*NPHS2*) had been found to play a significant role in SRNS, consisting of approximately 20 to 40% of familial and 10% of idiopathic childhood SRNS in different regions of the world (10–13). Podocin is composed of 383 amino acids localizing specifically from the insertion of the slit diaphragm (SD) to the podocyte cytoplasm (7, 14). It is required for the structural organization and regulation of the glomerular filtration barrier by interacting with other important SD molecules such as nephrin and CD2-associated protein (CD2AP) (15–18). rs61747728 (p.R229Q, G686A) is one of the most commonly reported variants in podocin. A study conducted by Tsukaguchi et al. (19) found that p.R229Q caused a decrease in the ability of podocin to bind to nephrin and was usually associated with secondary *NPHS2* mutation, which enhanced the susceptibility to develop FSGS. Meanwhile, some studies reported that heterozygous p.R229Q polymorphisms were associated with SRNS when compared to a healthy population (20, 21). Furthermore, p.R229Q-podocin was found to be related with microalbuminuria in the general population (10). In contrast with these findings, p.R229Q was found not associated with SRNS or urinary albumin-creatinine ratio (ACR) in some other studies performed on White or Black individuals (10, 20, 22). These controversial findings might be due to underpowered study design or different study populations. In addition, few studies were performed on Asian population although the prevalence of SRNS was relatively higher in Asians and African Americans (3, 23, 24). The aim of this study was to investigate the association between p.R229Q and FSGS/SRNS. We first conducted a screening study on our cohort composed of adult patients who were of Chinese descent and with FSGS/SRNS. To enhance the power of this study, we performed a meta-analysis by pooling data driven from our cohort and from bodies of literature obtained from public databases.

## Methods

### Screening Study

#### Patients and Controls

Inclusion criteria were as follows: (1) age between 18 to 65 years old, (2) newly diagnosed nephrotic syndrome without previous usage of immunosuppressive agents within 1 month before the recruitment, and 3) renal biopsy-proved FSGS, minimal change disease (MCD), or IgA nephropathy (IgAN). We included patients with IgAN treated with full-dose prednisone as their initial therapy. SRNS was defined as less than 50%reduction in urine protein compared to baseline or urine protein higher than 3.5 g/d after treatment with a full dose of prednisone (1 mg/kg, maximum 80 mg/day) for at least 12 weeks. Healthy controls were defined as having serum creatinine bellow 100 umol/L without proteinuria or hematuria by urine routine test. All the individuals recruited in this study self-reported as having a Chinese Han origin. Informed consent was obtained from all the study participants before enrollment. The study protocol was reviewed and approved by Ruijin Hospital Human Research Ethics Committee.

#### Mutation Analysis

Genomic DNA was extracted and purified from peripheral leukocytes in peripheral blood cells of all the included subjects. All the eight coding regions, exon-intron boundaries, 5′UTR, and 3′UTR of *NPHS2* were amplified by polymerase chain reaction (PCR) (7). Primers were designed with the primier5 software or based on published information (19) (Supplementary Table 1). The PCR products were sequenced by Invitrogen. Sequence chromatograms were analyzed with the SEQUENCHER™ software (Gene Code Corp., Ann Arbor, MI, United States) by comparing with the reference sequence of *NPHS2* downloaded from NCBI^1^.

### Systemic Review and Meta-Analysis

#### Search Strategy and Inclusion Criteria

Relevant studies published until 1 March 2021 were searched through electronic databases of PubMed, SCOPUS, Cochrane Library, and Web of Science and using the search terms “R229Q,” “*NPHS2*,” “rs61747728,” “686G > A,” and “Arg229Gln.” Additional studies were also searched by reviewing the references cited in the retrieved articles.

Studies eligible for inclusion in our meta-analysis had to fulfill the following criteria: (1) studies discussing about the association between p.R229Q and SRNS (or FSGS) (e.g., SRNS group vs. control group or FSGS group vs. control group), (2) original data of genotype frequencies available, (3) SRNS was defined as patients who do not achieve complete remission within 12 weeks of glucocorticoid treatment, (4) early onset was defined as onset age ≤18 years, late onset was defined as onset age >18 years. Studies were excluded from our analysis if: (1) there was no control group, (2) genotype data were not available, (3) they were a duplicate of previous publication, and (4) they were reviews, editorials, or unpublished reports.

#### Data Extraction

All the data were extracted independently by two reviewers (Qiongxiu Zhou and Xiaoyan Zhang) according to a standard protocol. The following information was extracted from the eligible studies: first author’s surname, year of publication, ethnicity of subjects, sample size, age, and distribution of p.R229Q genotype and allele frequencies in case and control groups.

### Statistical Analyses

In the current meta-analysis, the associations between p.R229Q and FSGS/SRNS was analyzed by calculating the pooled odds ratios (ORs) and their 95% confidence intervals (CIs) using a random-effects model. Three genetic models were used for the association study: the allelic model (A vs. G), the dominant model (GA + AA vs. GG), and the recessive model (AA vs. GG + GA) (25, 26). Subgroup analyses were conducted according to subject ethnicity and age of onset.

Hardy-Weinberg Equilibrium (HWE) was tested by chi-square test (χ^2^) for goodness of fit to compare the observed and expected genotype frequencies with controls using a previous meta-analysis as reference, and a *P*-value < 0.01 was considered as significant deviation from HWE. As deviation from HWE in subjects may bias the estimates of genetic effects in a meta-analysis, a sensitivity analysis was conducted by removing one study and recalculating the pooled OR and 95% CI to assess the stability of the results. Potential publication bias was estimated by Begg’s funnel plot. All statistical tests were performed with the STATA 11.0 software (Stata Corp., College Station, TX, United States). Two-sided *P* < 0.05 was considered statistically significant.

## Results

### Screening Study on the Present Cohort

In total, there were 61 patients with SRNS and 204 patients with FSGS recruited in the screening study. Among the 61 patients with SRNS, 46 (75%) had FSGS, 9 (15%) had MCD and 6 (10%) had IgAN; 4 of the 6 patients with IgAN had FSGS changes in renal pathologic findings. The baseline characteristics of patients with SRNS and FSGS enrolled are shown in Supplementary Table 2. We also recruited 100 healthy individuals as a control group. Four synonymous single nucleotide polymorphisms (SNPs), c.187A > G (p.G34G) in exon1, c.288C > T (p.S96S) in exon2, and c.954C > T (p.A138A) and c.1038A > G (p.L346L) in exon8 of *NPHS2*, were identified by Sanger sequencing among all the individuals recruited in the screening study. However, p.R229Q was absent in all the patients with SRNS and FSGS.

### Meta-Analysis

#### Searching Studies for Meta-Analysis

There were 21 studies investigating the association between p.R229Q and SRNS/FSGS, including the present study. A flow chart shows the literature search for relevant studies on the association between p.R229Q and SRNS/FSGS (Supplementary Figure 1). The studies contained 2,489 cases and 6,004 controls with 12 comparisons on Caucasians, 6 on Asians, and 3 on Africans. Characteristics of these studies evaluating the association between R229Q polymorphism and SRNS/FSGS risk are shown in Table 1. The frequency distributions of genotypes were consistent with HWE.

**TABLE 1:** Characteristics of studies evaluating the association between R229Q polymorphism and SRNS/FSGS risk.

Comparison	Study	Onset age	Race	No. of case /control	Genotype frequencies (GG/GA/AA)		
					case	control	P (HWE)
SRNS vs. control	Mishra (40)	early-onset	Asian	20/50	14/6/0	37/13/0	0.29
	Fotouhi (41)	late-onset	Asian	25/35	25/0/0	35/0/0	1.00
	Abid (42)	early-onset	Asian	145/100	143/0/2	99/1/0	0.00
	Reiterova (43)	late-onset	Caucasian	36/300	32/4/0	270/29/1	0.81
	Tsygin (44)	early-onset	Caucasian	59/15	48/11/0	13/2/0	0.78
	Santin (45)	all age	Caucasian	139/227	126/12/1	218/9/0	0.76
	Megremis (46)	early-onset	Caucasian	22/100	20/2/0	97/3/0	0.88
	Machuca et al. (36)	all age	Caucasian	214/308	179/32/3	292/16/0	0.64
		all age	Caucasian	47/70	31/16/0	69/1/0	0.95
		all age	African	40/95	35/3/2	88/7/0	0.71
		all age	NA	34/14	31/3/0	14/0/0	1.00
	Mao et al. (37)	early-onset	Asian	22/30	22/0/0	30/0/0	1.00
	Weber et al. (20)	early-onset	Caucasian	319/320	294/18/7	308/12/0	0.73
	Ruf et al. (10)	early-onset	Mix	190/80	177/11/2	71/9/0	0.59
	Caridi et al. (32)	early-onset	Caucasian	120/100	110/7/3	95/5/0	0.80
	Karle et al. (31)	early-onset	Caucasian	31/100	28/3/0	94/6/0	0.76
	Zaki (47)	early-onset	African	22/53	7/15/0	51/2/0	0.27
	Baylarov (48)	early-onset	Asian	21/21	20/0/1	21/0/0	0.00
	Our study (2022)	late-onset	Asian	61/100	61/0/0	100/0/0	1.00
FSGS vs. control	Fotouhi (43)	late-onset	Asian	25/35	25/0/0	35/0/0	1.00
	Abid (42)	early-onset	Asian	48/100	47/0/1	99/1/0	0.96
	Reiterova (43)	late-onset	Caucasian	50/300	44/6/0	270/29/1	0.81
	Tsygin (44)	early-onset	Caucasian	59/15	48/11/0	13/2/0	0.78
	Tonna et al. (30)	all age	Mix	371/2596	330/40/1	2420/169/7	0.03
	McKenzie (50)	late-onset	African	247/634	242/5/0	618/16/0	0.75
		late-onset	Caucasian	129/271	117/12/0	250/21/0	0.51
	He (50)	late-onset	Caucasian	63/54	58/5/0	51/3/0	0.83
	Aucella et al. (34)	late-onset	Caucasian	33/124	30/3/0	117/7/0	0.75
	Tsukaguchi et al. (19)	late-onset	Mix	91/257	NA	NA	NA
	Our study (2022)	late-onset	Asian	204/100	204/0/0	100/0/0	1.00

#### Association Between p.R229Q and SRNS/FSGS

**FIGURE 1:**
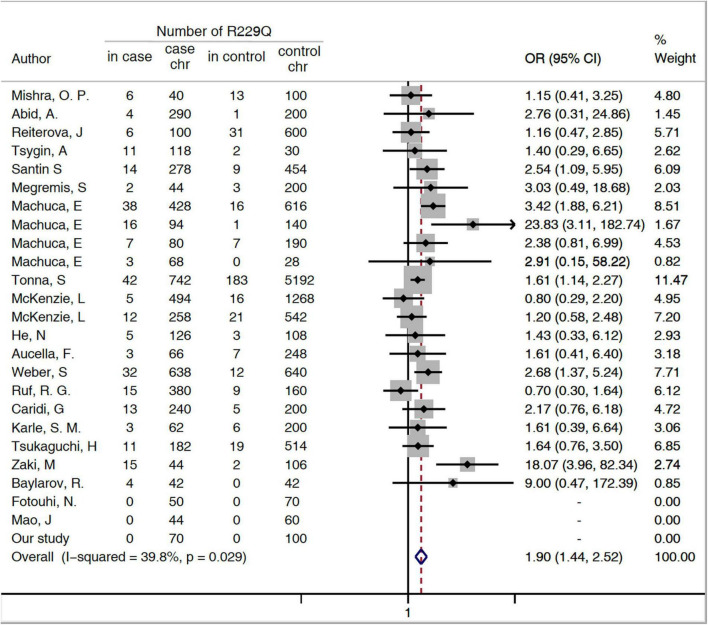
Forest plots of meta-analysis of association between p.R229Q and FSGS/SRNS in the allelic model. Chr, chromosome; CI, confidence interval; OR, odds risk; SRNS, steroid-resistant nephrotic syndrome; and FSGS, focal segmental glomerular sclerosis.

The minor allele frequency (MAF) (A allele) of p.R229Q was.06 in the patients with SRNS/FSGS and.03 in the healthy controls. An increased risk for p.R229Q in SRNS/FSGS was confirmed by the allelic model (OR = 1.9, 95% CI 1.44-2.52, *P* < 0.001, Figure 1), the recessive model (OR = 3.9, 95% CI 1.56-9.77, *P* = 0.004, Supplementary Figure 2) and the dominant model (OR = 1.91, 95% CI 1.37-2.73, *P* < 0.001, Supplementary Figure 3).

**FIGURE 2:**
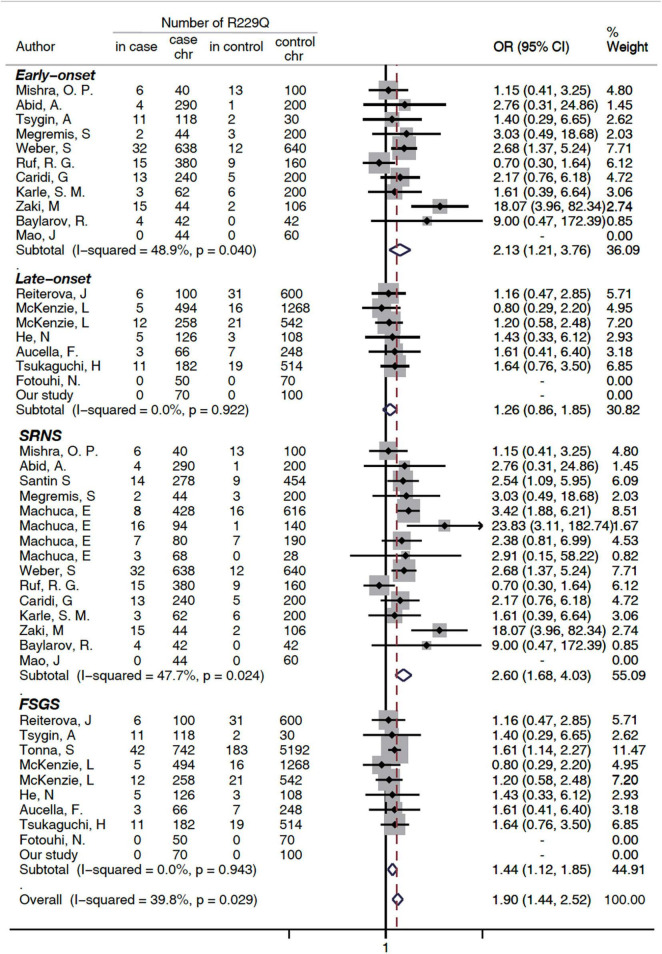
Subgroup analysis of association between FSGS/SRNS and the p.R229Q variant based on age of onset and disease types (allelic model). CI, confidence interval; OR, odds risk; chr, chromosome; SRNS, steroid-resistant nephrotic syndrome; and FSGS, focal segmental glomerular sclerosis. SRNS, steroid-resistant nephrotic syndrome; FSGS, focal segmental glomerular sclerosis; HWE, Hardy Weinberg Equilibrium; NA, not available (i.e., not stated).

**FIGURE 3:**
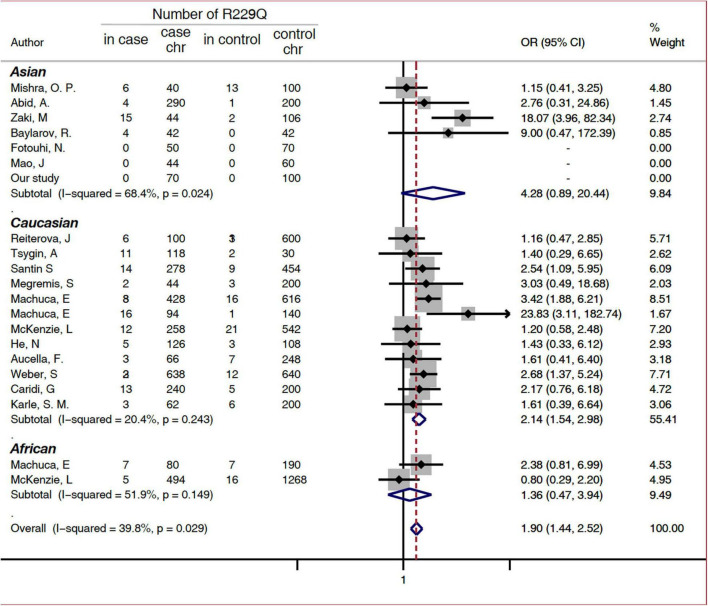
Subgroup analysis of association between FSGS/SRNS and the p.R229Q variant based on ethnicity (allelic model). CI, confidence interval; OR, odds risk; chr, chromosome; SRNS, steroid-resistant nephrotic syndrome; and FSGS, focal segmental glomerular sclerosis.

A subgroup analysis was performed based on disease phenotype: SRNS or FSGS. A significant increased risk of SRNS and FSGS in patients with R229Q was determined based on the allelic model (OR = 2.6, 95% CI 1.68-4.03, *P* = 0.024 and OR = 1.44, 1.12-1.85, 95%CI *P* <0.001, respectively, Figure 2). When all the patients were stratified by ethnicity, significant risks were found among Caucasians either in the allele-based analysis (OR = 2.14, 95% CI 1.54-2.98, *P* < 0.001, Figure 3) or in the genotype-based analysis (dominant model: OR = 2.11, 95% CI: 1.48-3, *P* < 0.001; recessive model: OR = 6.56, 95% CI 1.69-25.5, *P* = 0.007). However, no association was found in the African and Asian populations. In the subgroups stratified by onset age, the p.R229Q variant increased the risk of SRNS among the early-onset patients in the allelic (OR: 2.13, 95% CI = 1.21-3.76, *P* = 0.009, Figure 2), recessive (OR = 4.85, 95% CI = 1.25-18.81, *P* = 0.02), and dominant (OR: 2.09, 95% CI = 1.04-4.21, *P* = 0.04) models, while no significant association was found in all the three genetic models among the late-onset patients.

#### Sensitivity Analysis

In this meta-analysis, significant heterogeneity (I^2^ = 52.1%, *P* = 0.003) was observed in the included dominant model, so a sensitivity analysis performed. The combined ORs were similar with one another, with a narrow range from 1.72 (95% CI: 1.32-2.26) to 2.08 (95%CI: 1.49-2.91) (Supplementary Figure 4). It indicated that our results were stable.

#### Publication Bias

The publication bias of the individual studies was evaluated by Begg’s test. No significant difference was found (*P* = 0.15). It indicated that there was no significant publication bias in our meta-analysis (Supplementary Figure 5).

## Discussion

*NPHS2* mutations were initially described in patients with NS from birth to 6 years of age (20, 27), while they were infrequently detected in children with non-familial SRNS (28, 29). A study on adults with FSGS/SRNS has not been fully performed. In our study, we examined the frequency of *NPHS2* mutation in a cohort of adult Chinese patients with SRNS or FSGS. Four known synonymous variations (G34G, S96S, A318A, and L346L) were identified. However, the distribution of genotypes among our patients and the controls were similar, which suggested that the SNPs did not increase the risk of SRNS or FSGS. We did not find non-synonymous variations in *NPHS2*, and no p.R229Q was observed in patients with either SRNS or FSGS. It suggested that adult-onset *NPHS2* mutation was rare in Chinese patients, and that p.R229Q had no effect on the patients, at least in our population.

In the current meta-analysis, we found that the frequency of the p.R229Q variant was significantly higher in the patients with SRNS/FSGS than in the healthy populations in the allelic (OR = 1.9, 95% CI 1.44-2.52), dominant (OR = 1.91, 95% CI 1.37-2.73), and recessive (OR = 3. 0, 95% CI 1.56-9.77) models. Furthermore, it was significant in the subgroups of either SRNS (OR 2.6, *P* = 0.024) or FSGS (OR 1.44, *P* <0.001). As FSGS was a common cause of SRNS in both children and adults, we could hypothesize that p.R229Q plays a pathogenic role in FSGS and SRNS. These findings were consistent with previous studies, suggesting that the p.R229Q allele may be a disease-causing variant, which could enhance the susceptibility of FSGS, and FSGS patients with podocin mutations were more likely to be p.R229Q in heterozygous state with one pathogenic mutation (10, 19, 20, 30–34).

The results from our meta-analysis were different from Lu Lu’s study (35), which reported no association between p.R229Q and FSGS. First, we recruited more cases, with 2,489 patients with SRNS/FSGS and 6,004 controls, and there were more comparisons on different ethnicity, with 12 comparisons on Caucasians, 6 on Asians, and 3 on Africans. Secondly, Lu L’s study excluded the studies that all variant individuals are compound heterozygotes, considering that the excessive possible affecting SNPs in the *NPHS2* gene related to FSGS was difficult to identify. We agree with this concern. Therefore, in our study, we constructed genetic models, the allelic (A vs. G), dominant (GA + AA vs. GG), and recessive (AA vs. GG + GA) models, to explain the allele frequency and genotype-phenotype correlation of p.R229Q in these studies. We concluded that p.R229Q might play a pathogenic role in developing SRNS/FSGS in the state of compound heterozygotes (36).

Additionally, we found that the frequency of p.R229Q was various throughout different populations. The frequency was higher in Caucasians than in Asians and Africans (36). For the Caucasians, p.R229Q increased the risk of FSGS/SRNS (allelic model: OR = 2.14, 95% CI 1.54-6.64; dominant model: OR = 2.11, 95% CI 1.48-3; recessive model: OR = 3.9, 95% CI 1.56-9.76). However, the associations with other populations were not significant. In our screening study, we did not find the p.R229Q variant in Chinese patients with adult-onset SRNS and FSGS, which was in agreement with a previous study conducted on Chinese patients with childhood-onset SRNS (37). This suggested that p.R229Q allele distribution in different races was uneven, although one explanation for this discrepancy might be that relatively small studies and low frequencies of p.R229Q in these populations could have limited the statistical power for analysis. Besides, significant results were also observed in patients with early-onset FSGS/SRNS in the allelic (OR: 2.13, 95% CI 1.21-3.76), dominant (OR: 2.11, 95% CI 1.48-3) and recessive (OR: 6.56, 95% CI 1.69-25.5) models compared with the healthy controls. We could hypothesize that p.R229Q was more likely to act as a disease modifier to increase the risk for patients with early-onset FSGS/SRNS.

Even though many studies and meta-analyses had been conducted on the genetic role of the p.R229Q variant in the previous years, most of them were performed on Caucasians. Limited studies were available on African and Asian populations. This limited our ability to reach a strong conclusion and investigate a potential function on the basis of race. Moreover, p.R229Q was recently discovered pathogenic only when trans-associated to specific mutations, and this could not be analyzed by the current meta-analysis (38, 39).

In conclusion, our study indicated that *NPHS2* mutations were rare in Asian patients with sporadic adult-onset FSGS/SRNS and p.R229Q was undetectable in our cohort. *NPHS2* p.R229Q may play an important role in enhancing susceptibility to FSGS/SRNS, especially in Caucasian and early-onset patients. Further studies and international multi-ethnicity approaches are needed to distinguish a pathogenic and benign p.R229Q genotype-phenotype correlation for clinical assessment.

## Data Availability Statement

The original contributions presented in this study are publicly available. This data can be found here: GenBank, accessions ON470453 - ON470576.

## Ethics Statement

The studies involving human participants were reviewed and approved by Ruijin Hospital Human Research Ethics Committees. The patients/participants provided their written informed consent to participate in this study.

## Author Contributions

QZ and XZ completed the screening test and extracted all the data that were needed for the meta-analysis. QZ and QW drafted the original manuscript. YL, JT, and XH helped in polishing the manuscript and were responsible for the figures. JX and NC created the overall design of this study. HS, PS, and HR revised the article. All authors contributed to the article and approved the submitted version.

## Conflict of Interest

The authors declare that the research was conducted in the absence of any commercial or financial relationships that could be construed as a potential conflict of interest. The reviewer FL declared a shared parent affiliation with the authors JX, QW, JT, XH, HS, XZ, YL, PS, HR, and NC to the handling editor at the time of review.

## Publisher’s Note

All claims expressed in this article are solely those of the authors and do not necessarily represent those of their affiliated organizations, or those of the publisher, the editors and the reviewers. Any product that may be evaluated in this article, or claim that may be made by its manufacturer, is not guaranteed or endorsed by the publisher.

## Footnotes

1
http://www.ncbi.nlm.nih.gov/
