# Supplementary material for: Association Between NPHS2 p.R229Q and Focal Segmental Glomerular Sclerosis/Steroid-Resistant Nephrotic Syndrome
Source: Front Med (Lausanne). 2022 Jul 22;9:937122. doi: 10.3389/fmed.2022.937122 (PMC9354893; doi:10.3389/fmed.2022.937122)
Supplement: Supplementary file 1 [file Data_Sheet_1.docx]

**Supporting information**

Supplementary Table1. PCR primers for amplifying NPHS2 gene.

|  | Forward primer | Reverse primer |
| --- | --- | --- |
| Exon1 | GCAGCGACTCCACAGGGACT | TCAGTGGGTCTCGTGGGGAT |
| Exon2 | AGGCAGTGAATACAGTGAAG | GGCCTCAGGAAATTACCTA |
| Exon3 | TTCTGGGAGTGATTTGAAAG | TGAAGAAATTGGCAAGTCAG |
| Exon4 | AAGGTGAAACCCAAACAGC | CGGTAGGTAGACCATGGAAA |
| Exon5 | CATAGGAAAGGAGCCCAAGA | TTCAGGCATATTGGCCATTA |
| Exon6 | CTCCCACTGACATCTGA | AATTTAAAATGAAACCAGAA |
| Exon7 | CTAAATCAATGGCTGCACCACC | TTCCTAAAGGGCAGTCTGG |
| Exon8 | GGTGAAGCCTTCAGGGAATG | TTCTATGGCAGGCCCCTTTA |

Supplementary Table2. Clinical features of including patients in our study.

|  | SRNS(n=61) | FSGS(n=204) |
| --- | --- | --- |
| Gender (Female/Male) | 53:28 | 123:81 |
| Age at onset (years) | 34 | 30.5 |
| Proteinuria (g/24h) | 5.2±3.4 | 2.2±1.4 |
| Serum Creatinine (umol/L) | 121.0±78.5 | 141.0±79.5 |
| Renal biopsy  (FSGS:MCD:IgAN) | 46:9:6 | _ |

SRNS, steroid-resistant nephrotic syndrome; FSGS, focal segmental glomerular sclerosis; MCD, minimal change disease; IgAN, IgA nephropathy.

Supplementary Figure1. Flow chart of studies selection. Flow chart shows literature search for relevant studies about the association between p.R229Q and SRNS or FSGS. SRNS= Steroid resistant nephrotic syndrome; FSGS=Focal segmental glomerular sclerosis.


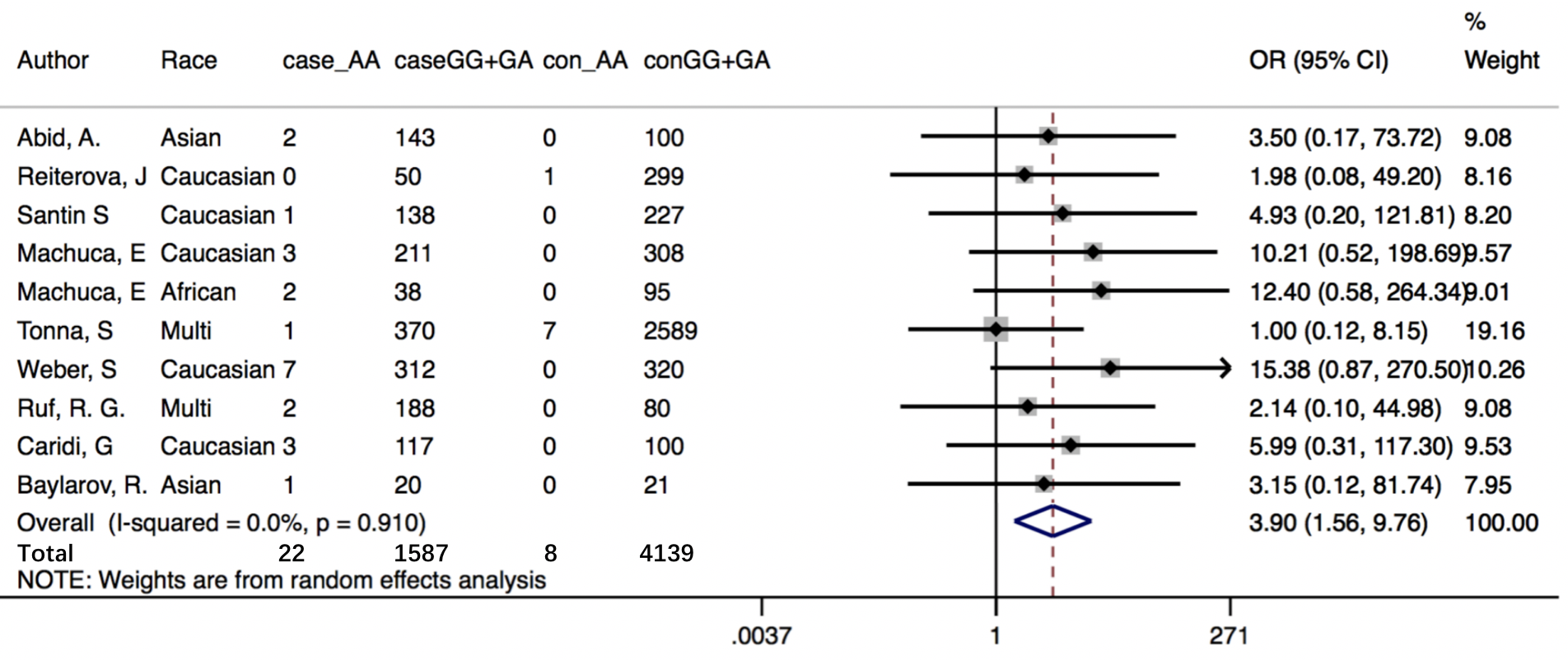


Supplementary Figure2. Forest plots of meta-analysis of association between p.R229Q and FSGS/SRNS in recessive model. CI=confidence interval; OR=odds risk. SRNS=Steroid resistant nephrotic syndrome; FSGS= Focal segmental glomerular sclerosis; Con=Control.


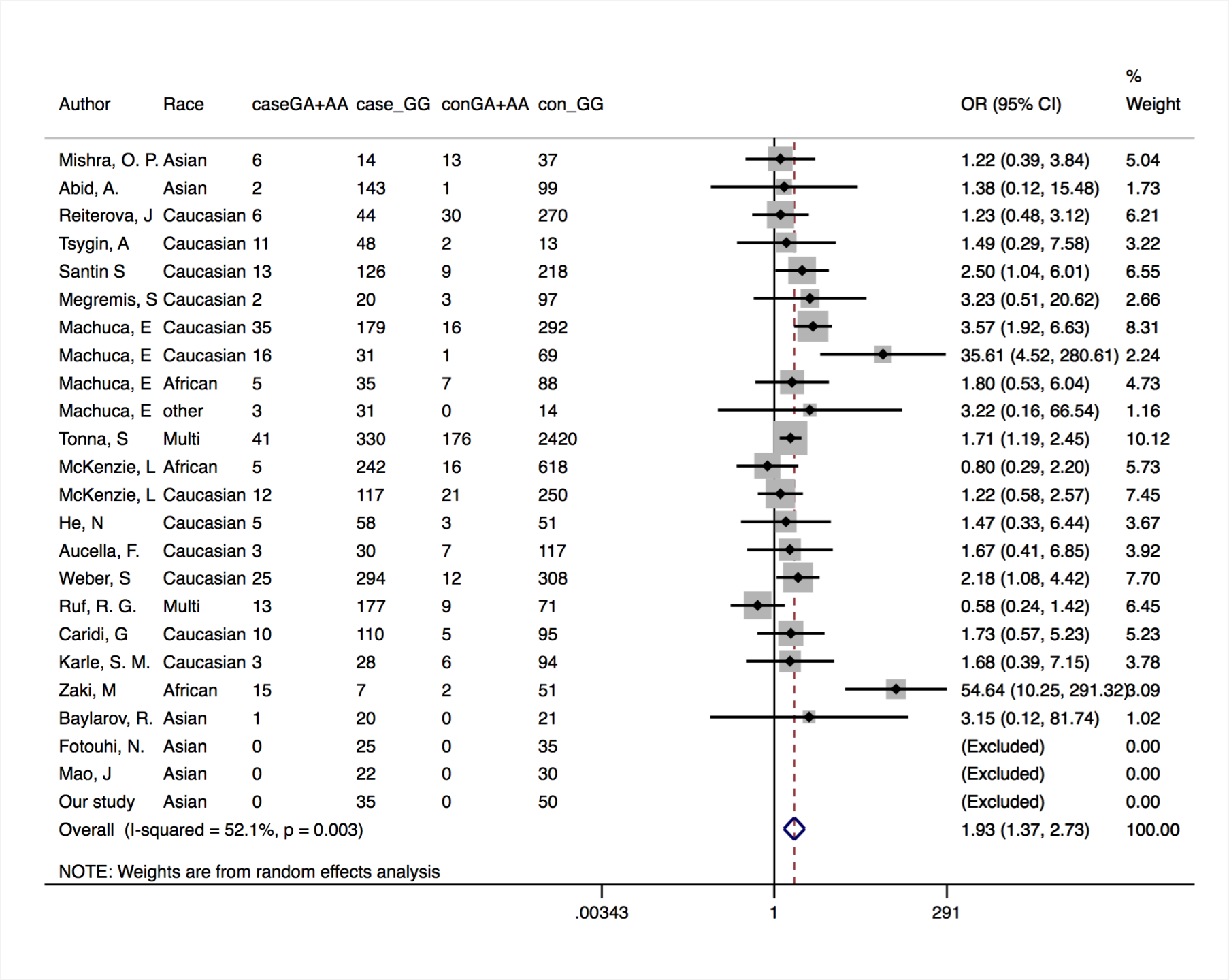


Supplementary Figure3. Forest plots of meta-analysis of association between p.R229Q and FSGS/SRNS in dominant model. CI=confidence interval; OR=odds risk. SRNS=Steroid resistant nephrotic syndrome; FSGS=Focal segmental glomerular sclerosis; Con=Control


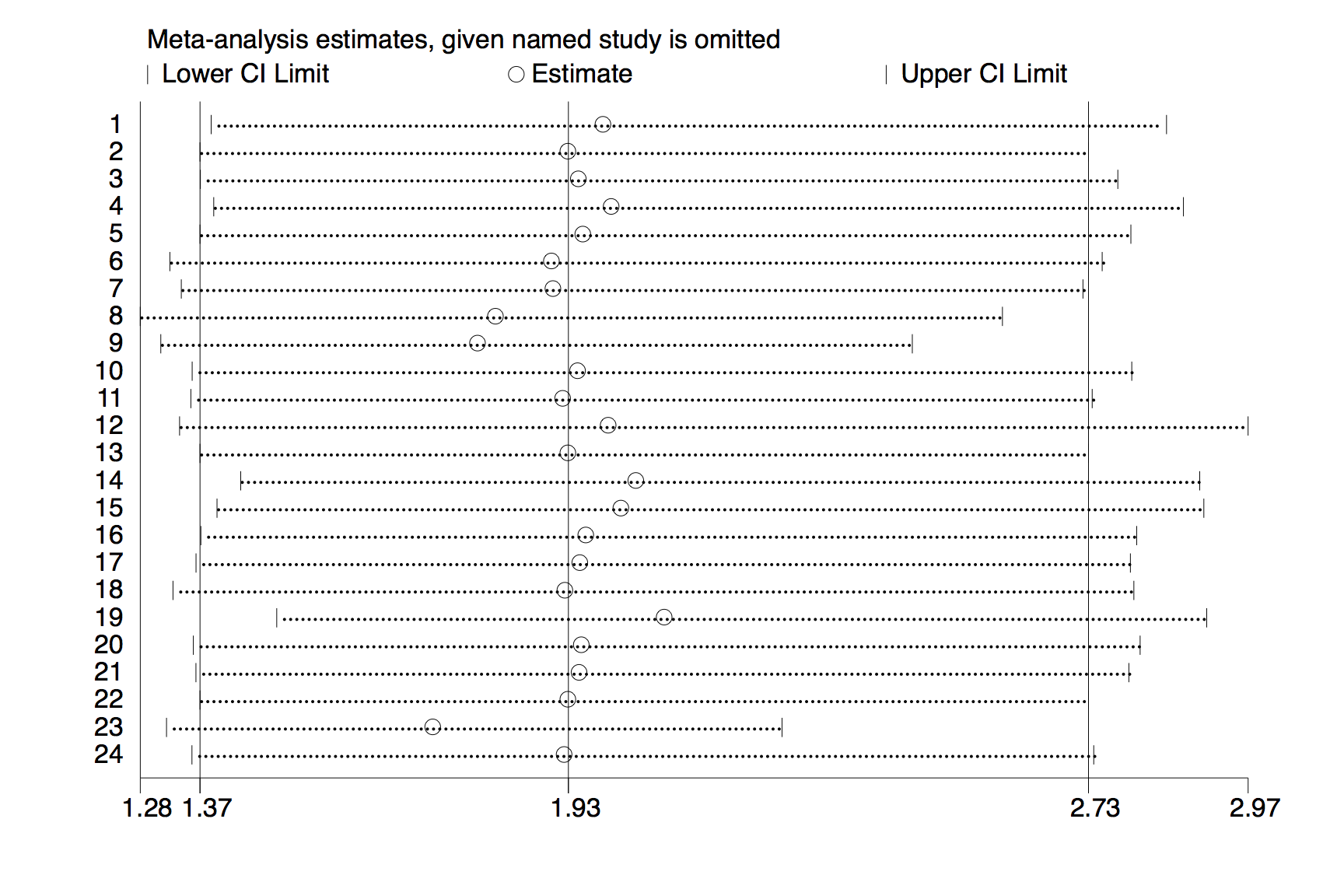


Supplementary Figure4. Sensitivity analysis was performed in the studies of SRNS/FSGS and healthy controls (dominant model). By removing one study and recalculating the pooled OR and 95%CI to assess the stability of the results, the combined ORs were similar with one another, with a narrow range from 1.72 (95%CI: 1.32-2.26) to 2.08(95%CI:1.49-2.91). CI=confidence interval; SRNS= Steroid resistant nephrotic syndrome.


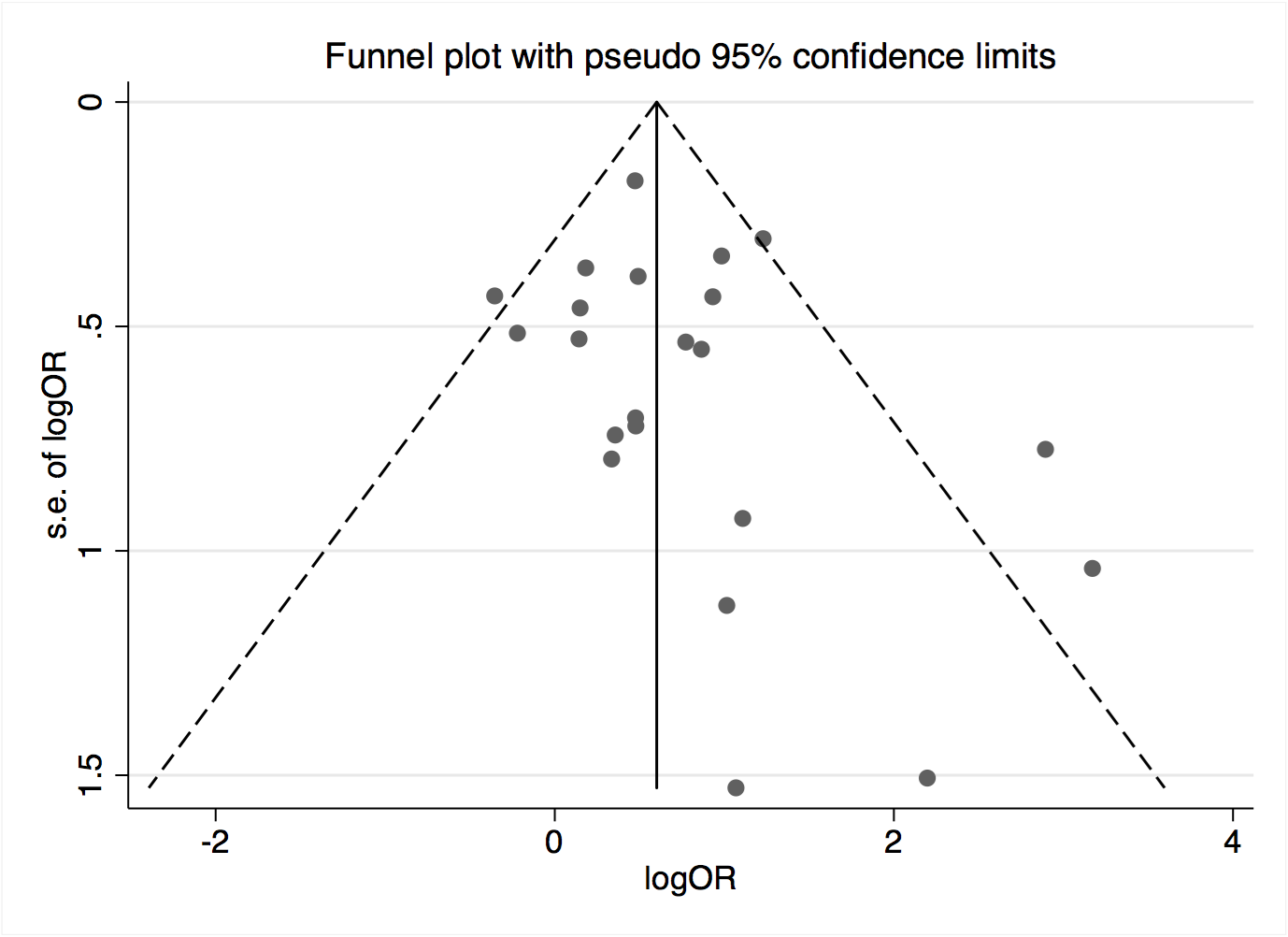


Supplementary Figure5. Funnel plots were constructed for p.R229Q meta-analysis by assessing symmetry to visually evaluate the publication bias and no significant difference was found. P=0.15. OR=odds risk; SRNS= Steroid resistant nephrotic syndrome; FSGS= Focal segmental glomerular sclerosis.
